# Preparation of Super-Flexible Silica Aerogel and Its Application in Oil–Water Separation

**DOI:** 10.3390/gels9090739

**Published:** 2023-09-12

**Authors:** Linghan Li, Tianci Xu, Faping Zhang, Chunhua Du, Song He

**Affiliations:** School of Safety Science and Emergency Management, Wuhan University of Technology, Luoshi Road 122, Wuhan 430070, China; nuonuomi@whut.edu.cn (L.L.); 307833@whut.edu.cn (T.X.); 307843@whut.edu.cn (F.Z.); 303297@whut.edu.cn (C.D.)

**Keywords:** flexible silicon aerogel, adsorption separation research, sol-gel method, oil/water separation test, recyclability

## Abstract

Using silica as the precursor, and methyltrimethoxysilane and dimethyldimethoxysilane as the silicon sources, a super-flexible hydrophobic lipophilic gel solid was prepared via hydrolysis, drying, solvent replacement, and atmospheric-pressure drying. The characterization test showed that the sample had good flexibility, hydrophobicity, an amorphous structure, and a hydrophobic contact angle of 137°. Through the adsorption separation experiment, it was concluded that the adsorption separation rate of aerogel to oil substances is related to the viscosity of the oil substances. The hydrophobic and oleophilic properties of flexible silicon aerogel materials can be applied to many aspects, such as crude oil leakage and kitchen waste oil recovery, with broad future development prospects and great research significance.

## 1. Introduction

Silica aerogel is an emerging porous material that has been widely used and applied due to its high porosity and large specific surface area. However, the mechanical properties of silica aerogels are very poor. Due to the drying requirements in the preparation process, aerogels often exhibit low strength, poor flexibility, and poor integrity. This greatly limits the practical application of silica aerogels. In general, the preparation methods of silica aerogels include the sol-gel method and hydrothermal method. The sol-gel method refers to a method where inorganic compounds are hydrolyzed and condensed in a solution to form a gel with a spatial network structure and porous structural materials are obtained after removing the solvent [1]. The process of preparing aerogels by this method includes hydrolysis, drying, solvent replacement, atmospheric pressure drying, etc. Although supercritical drying technology is widely used in actual manufacturing, it has high requirements for pressure control, which leads to high equipment costs [2]. And because of the use of high-temperature and high-pressure processes, the operation is dangerous. In addition, the drying medium is mostly an alcohol–organic solvent, which is flammable and prone to danger. Compared with supercritical drying, ambient pressure drying is safe, low-cost, and simple to operate. The principle of atmospheric drying is to modify the gel skeleton with hydrophobic groups to avoid the combination of silicon hydroxyl groups on the surface of the gel pores and improve the elasticity. At the same time, a low-surface-tension liquid is used to replace the original high specific surface area of the gel. Water or ethanol can be directly dried under atmospheric pressure to obtain aerogel materials with excellent properties. However, atmospheric-pressure drying has high requirements for pretreatment, and during the drying process, shrinkage and cracking are prone to occur due to the very large surface tension of the solid–liquid interface in the pores. Therefore, only the production enterprises with mature atmospheric processes have a competitive advantage; otherwise, the cost advantage is not prominent. The disadvantage is that the solvent still has a certain surface tension or may cause structural collapse [3,4,5].

In recent years, many scholars have explored the preparation of new silica aerogels to overcome the defects of traditional silica aerogels [6]. For example, Lynn A. Capadona et al. used the polymerization of a di-isocyanate with an amine-modified surface of a sol-gel-derived mesoporous silica network to crosslink the nanoparticles of the silica skeleton. and reinforce its fragile framework [7]. Gen Hayase et al. prepared new aerogels using trifunctional alkoxysilane (MTMS) and difunctional alkoxysilane dimethyldimethoxysilane (DMDMS) as precursors. Unlike aerogels using tetrafunctional and trifunctional alkoxysilanes in the past, this new aerogel is softer and more elastic [8]. Guoqing Zu et al. synthesized a new polyorganosiloxane aerogel with superhydrophobicity, high elasticity, and high bendability based on polyvinyl-poly (dimethylsiloxane) (PVPDMS)/polymethylsilsesquioxane (PMSQ), which was synthesized via a radical polymerization/co-curing strategy [9].

Aerogels have been honored as one of the top ten materials that have changed the world by *Science*. Due to their hardness, minimum density, extremely small pore size, and extremely low thermal insulation coefficient, they have been applied in various fields such as the petrochemical, environmental protection, construction, and military industries. Since the creation of aerogel in 1931, it has undergone four industrializations and set 15 Guinness world records for its excellent properties. Aerogels include silica, carbide, nitride, organic, carbon, biomass, composite, and other aerogels. At present, the most mature aerogel is silica aerogel. Compared with other aerogel materials, the raw materials of SiO_2_ aerogel are abundant; its manufacturing process is simple and easy to control [10]. And its market size has also increased significantly. At present, 56% of the downstream demand structure of aerogel comes from oil and gas projects, 18% is used for industrial insulation, 9% is used for building construction, and 8% is used for transportation. In 2026, 14% will be used for construction, and the demand in the transportation sector will increase to 13%. At present, many research institutions have reported that aerogels will become an ‘accelerator‘ for the rapid development of science and technology in the future.

Silica aerogels have wide application prospects and significance in thermal insulation, optics, medicine, electricity, and other aspects. Due to its high porosity and large surface area, silica aerogel has good adsorption capacity, which makes it widely used in wastewater treatment, air purification, and other environmental fields. On the other hand, silica aerogel, as a solid material composed of ultrafine particles, is characterized by small particle size and low density, so it can float on the sea [11]. In the treatment of offshore crude oil leakages, various traditional separation methods have their limitations: oil tanker treatment recycles a large amount of oil, but its treatment range is limited by its volume limitation and oil slick spread area, the recovered oil contains a large amount of water, and the separation efficiency is low. Chemical reagent treatment can dissipate oil slicks in a large area, but it has obvious side effects on the surrounding ecology. Burning treatment causes air pollution and wastes a lot of energy. The ultra-soft silica aerogel studied in this project shows an advantage in solving this problem because of its low cost, high oil–water separation efficiency, simple operation, environmental protection, and many other advantages [12]. The use of aerogel to deal with the leakage of offshore crude oil overcomes the shortcomings of the need for secondary separation on oil tankers, and overcomes the shortcomings of environmental pollution caused by chemical reagent treatment and combustion treatment. It ensures an efficient oil–water separation rate and does not cause any pollution to the environment. It is characterized by energy savings and environmental protection [13,14,15,16]. At present, there are few research results in this field. Therefore, this study briefly explored the factors influencing the oil–water separation rate of aerogels.

## 2. Results and Discussion

### 2.1. Compositional Analysis

#### 2.1.1. XRD Analysis

**Figure 1:**
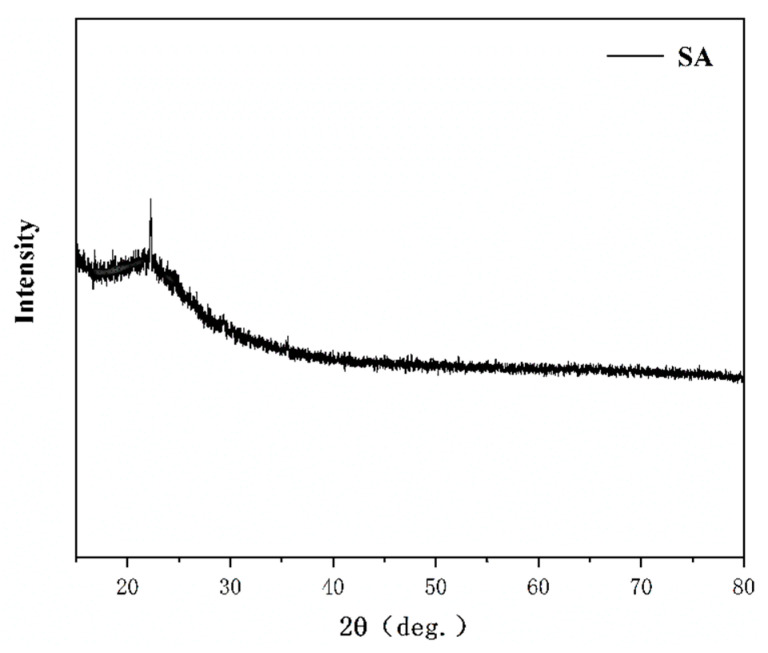
X-ray diffraction pattern of silica aerogel samples.

Silica aerogel is an amorphous and amorphous material. Figure 1 shows the results of the X-ray diffraction analysis. A broad peak centered at 2θ = 21.5 was observed. Therefore, it can be inferred that the flexible silica aerogel prepared in this study was amorphous and had no crystalline structure, which is consistent with the XRD analysis results for hydrophobic silica aerogel reported in the literature [17,18,19].

#### 2.1.2. Infrared Spectroscopic Analysis

**Figure 2:**
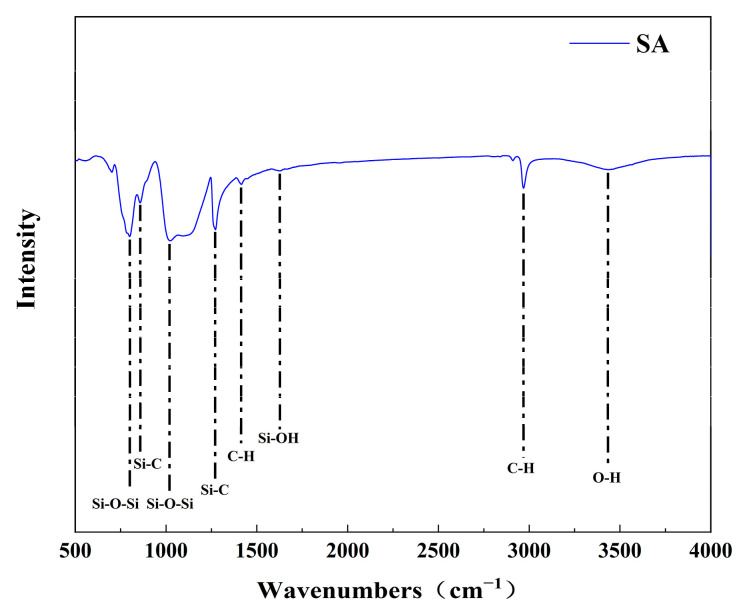
Infrared spectrum comparison of aerogels.

Infrared spectroscopy was used to analyze the functional groups contained in the substances, and the samples were analyzed via infrared spectroscopy. The results are shown in Figure 2. It can be concluded that the hydrophobic silica aerogel contained more abundant functional groups, which was reflected in the tensile vibration peak of Si–O–Si at 1000–1100 cm^−1^ and the deformation vibration absorption peak at 800 cm^−1^. The absorption peak near 1600 cm^−1^ is the tensile vibration peak of C–C. The absorption peaks at 1260 cm^−1^, 1410 cm^−1^, 2968 cm^−1^, and the Si–C bond at 856 cm^−1^ are caused by the in-plane and out-of-plane bending vibrations of CH_3_ on the Si–CH_3_ group [20,21,22]. The methyl group in the silica aerogel mainly comes from two silicon sources during the preparation process, and the methyl group (–CH3) makes it hydrophobic.

#### 2.1.3. XPS Analysis

**Figure 3:**
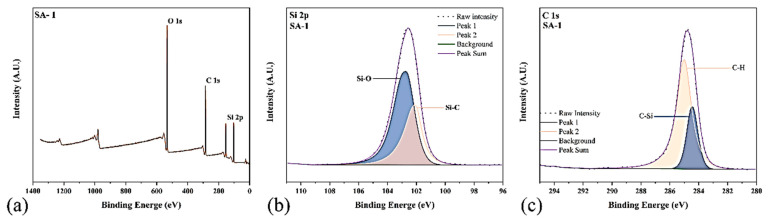
XPS (**a**–**c**) SA-1 of samples treated under different conditions.

XPS analysis can provide a clearer understanding of the proportion of atom numbers in a sample and the change in chemical bonds and be used analyze the main elements Si, O, and C in an aerogel [23]. According to the results in Table 1, the content of C element in hydrophobic silica aerogel was the largest, accounting for 40%, followed by Si element and O element. As shown in the figure, the silicon element is derived from the Si–C bond (binding energy of 102 eV) and the Si-O bond (binding energy of 103.5 eV). In addition, the C element is also derived from the C–H (285.7 eV) bond because it exists mainly in the form of methyl. The test results shown in Figure 3 and Table 1 show that the methyl groups in the two silicon sources were successfully grafted on the surface of the aerogel as hydrophobic groups, in agreement with the above test results.

### 2.2. Experiment Methodology

Flexible silica aerogels were prepared with the sol-gel method using methyltrimethoxysilane and dimethyldimethoxysilane as raw materials. After characterization and analysis, it was preliminarily concluded that the flexible silica aerogels studied had excellent hydrophobic properties. The adsorption characteristics of aerogels were studied via oil–water separation experiments [24].

**Figure 4:**
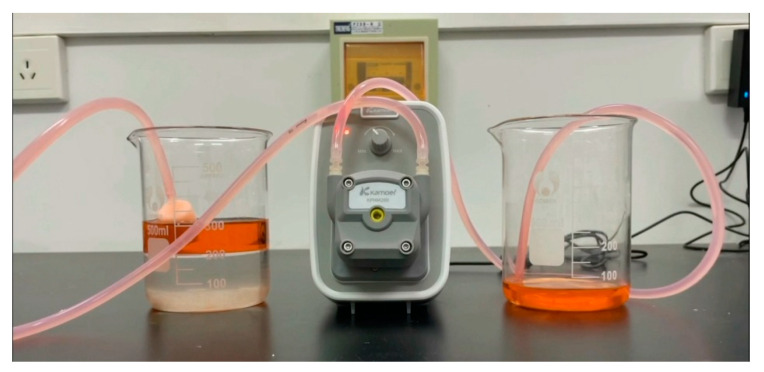
Flexible aerogel adsorption performance experiment.

First, the adsorption characteristics of the aerogels were studied. To facilitate observation, n-hexane treated with three Sudan red dyes was mixed with water in a ratio of 1:1, 100 mL each, and the mixed liquid appeared to have obvious stratification after configuration. The less-dense n-hexane was located above and below the water. By placing the connected aerogel in the oil three water mixture, it was found that the aerogel had been floating on the liquid surface, contacting with n-hexane and quickly adsorbing n-hexane to change color. The specifications of the silicone tube used in this experiment were 3.2 mm in inner diameter and 6.4 mm in outer diameter. The connection between the silicone tube and the aerogel was realized via an adapter and a beveled straw. The length of the connector connecting the silicone tube and the aerogel was 20 mm, the inner diameter was 3.2 mm, and the maximum diameter of the cone was 4.2 mm. After opening the peristaltic pump, n-hexane was adsorbed and separated by the aerogel from one end to the other end until all n-hexane was adsorbed and separated to the other end, as shown in Figure 4. Without closing the peristaltic pump, it could be observed that the aerogel had no liquid in the device after absorbing the stained n-hexane. This was due to the hydrophobic lipophilicity of the aerogel; that is, the aerogel could not adsorb water, which also confirmed the conclusion that the aerogel had hydrophobic properties [25]. The volume of n-hexane measured after closing the peristaltic pump was close to 100 mL, and the difference part was mainly residual in the inner wall of the silicone tube and the inside of the peristaltic pump, which could be approximately regarded as the complete adsorption of oil by the aerogel. It could be concluded that the loss of available oil substances was very small and negligible when oil–water separation was carried out with the aerogel. At the same time, it was observed that there was no stratification in the liquid at the output end obtained via adsorption separation; that is, there was no water, which also verified the previous conclusion.

In the adsorption experiment, the orange color of the aerogel gradually darkened due to the adsorption of dyed n-hexane. With the progress of oil–water separation, the oil substances in the pores of the aerogel gradually decreased, and the orange color of the aerogel became lighter and lighter. This represented the process of gradual filling of n-hexane in the internal voids of aerogels to the gradual replacement of n-hexane in the voids by air at the end of adsorption. After the experiment, it could be observed that the shape of the aerogel did not change significantly compared with that before the adsorption of n-hexane. The color changed from the initial white to dark orange after the adsorption of n-hexane. The flexible silica aerogel after the experiment was soaked in ethanol to remove the residual n-hexane and then dried under normal pressure. The aerogel returned to the previous white state. Since the adsorption separation of aerogels was based on the physical level, the theoretical analysis showed that the structure would not change significantly before and after adsorption [26]. Additionally, the adsorption separation experiment was carried out again on the aerogel after desorption drying, and the obtained data were not significantly different from those before desorption. So, we could conclude that the mechanical properties and hydrophobic properties of the flexible aerogel did not change significantly compared with those before the experiment, which indicated that the aerogel could be recycled in oil–water separation. 

Since the adsorption effect of aerogel came from its porous hydrophobic and lipophilic structural characteristics, and the diffusion rate of high-viscosity liquid inside the porous material was very slow [27], to explore whether the viscosity was the factor influencing the oil–water separation rate of the aerogel, an experiment was designed. Considering that the viscosity of oil substances is different in practical applications, to explore the application range of oil–water separation devices, this experiment was designed to explore the adsorption and separation rate of the flexible aerogel for oil substances with different viscosities. Four kinds of substances with large viscosity differences were selected: n-hexane (0.307 mPa·s), isopropanol (2.437 mPa·s), ethylene glycol (19.9 mPa·s), and vegetable oil (34 mPa·s) [28]. This was convenient to explore whether there was a significant change in the rate of oil–water separation of aerogels with different viscosity gradients.

**Figure 5:**
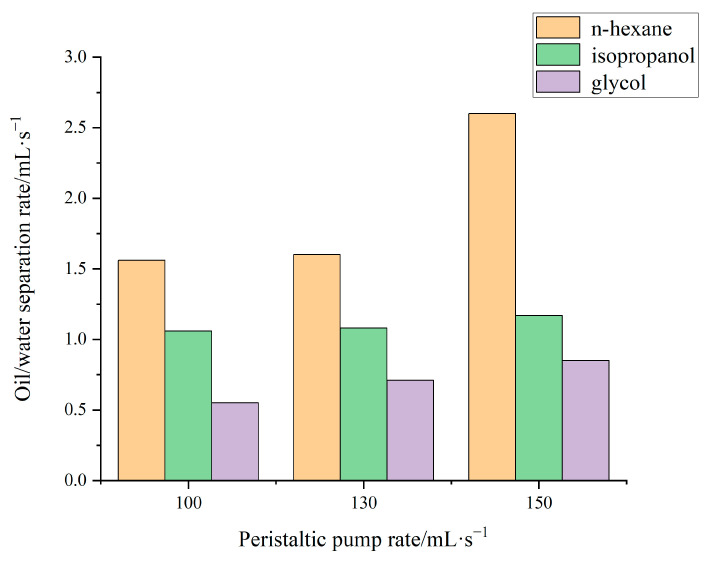
Relationship between oil substances and adsorption separation rate.

Through the control variable method, the rate of the peristaltic pump was unified, and three groups of peristaltic pumps with different rates were set up for experiments: 100 mL/min, 130 mL/min, and 160 mL/min, also known as the low-rate group, medium-rate group, and high-rate group. To reduce the error in the experiment and facilitate observation, the volume of the oil substance was set to 40 mL, and the time required to adsorb all the oil substances to complete the separation with the aerogel at each peristaltic pump rate was measured, and then the adsorption and separation speed of the aerogel for different-viscosity oil substances at each peristaltic pump rate were calculated [29]. To reduce the error, the timing starting point was taken as the moment when the peristaltic pump was opened after the pores of the aerogel were filled with oil. As the diameter of the silicone tube used in this experiment was large and the length was long, if the timing endpoint was taken as the time when the oil substance at the mixing end was completely adsorbed, the volume of the oil substance in the silicone tube was slightly larger [30]. There was likely no oil substance at the collection end when the adsorption end had been completely adsorbed, which was difficult to observe and inconvenient with regard to timing. Therefore, the timing end point was taken as the moment when there was no oil substance in the silicone tube; that is, the oil substance was adsorbed and completely separated into another container. Finally, the relationship between the adsorption separation rate of the flexible aerogel and the viscosity of the different oil substances is shown in Table 2 and Figure 5.

## 3. Conclusions

As a new solid material, aerogel has gradually developed from the original fragile material to a new material with many excellent characteristics such as super flexibility and superhydrophobicity. The present study combined silica aerogels with other organics to achieve oil–water separation by enhancing the hydrophobicity and elasticity of the aerogel, among other aspects. The major benefits of the material over the existing materials for oil–water separation are low cost and reusability. This is also consistent with Chen Ru et al.’s conclusion that ultra-flexible aerogels are reusable [31]. In addition, another promising finding is that silica aerogel has good development prospects in the field of oil–water separation due to its unique porous hydrophobic and lipophilic properties. In this study, a verification experiment of oil–water separation was designed, which showed that the aerogel had good oil–water separation characteristics and could be reused, which overcomes the limitations of traditional separation methods in the field of oil–water separation (especially in the treatment situation of offshore crude oil leakage). In addition, the experiment of the factors influencing oil–water separation rate of aerogel and viscosity explored in this paper also further illustrated that the viscosity of the oil affects the rate of oil–water separation of the aerogel, which generally shows a negative correlation trend. In the experiment, we chose the control variable method to carry out the experiment with the viscosity of the liquid and the speed of the peristaltic pump as the variables. Although the method is simple, the obtained results are very intuitive and effective. All in all, in this, the characterization properties and oil-water separation characteristics of aerogels were studied, and the following conclusions were drawn:

1. The flexible silica aerogels prepared from methyltrimethoxysilane and dimethyldimethoxysilane have good flexibility and excellent hydrophobic properties.
2. The flexible silica aerogel can be used for oil–water separation applications. After multiple adsorption–desorption cycles, its mechanical and adsorption properties remain unchanged, and it can be recycled.
3. Due to its excellent hydrophobic and lipophilic properties, flexible silica aerogels can be applied for environmental protection aspects such as offshore crude oil leakages and kitchen waste oil recovery.
4. The adsorption and separation rates of low-viscosity oil by aerogel are very considerable, but for high-viscosity oil, the rate is greatly reduced, but the effect is still acceptable.

## 4. Material and Methods

### 4.1. Materials

In this study, hydrophobic silica aerogels were prepared using the sol-gel method, using trimethoxymethylsilane and dimethyldimethoxysilane as silicon sources. The two Si sources are good for a more uniform microstructure. The presence of vinyl facilitates the elasticity and hydrophobicity of the aerogel [32]. The surface of the silica aerogel prepared with these silicon sources has a large number of methyl groups, which makes it have superior hydrophobicity and lipophilicity. The specific data support is provided in detail in Section 2. Its good mechanical properties and flexibility enable it to maintain its original structure after multiple press-fits, which provides the possibility for the reuse of aerogels to be mentioned later.

### 4.2. Sample Preparation

**Figure 6:**
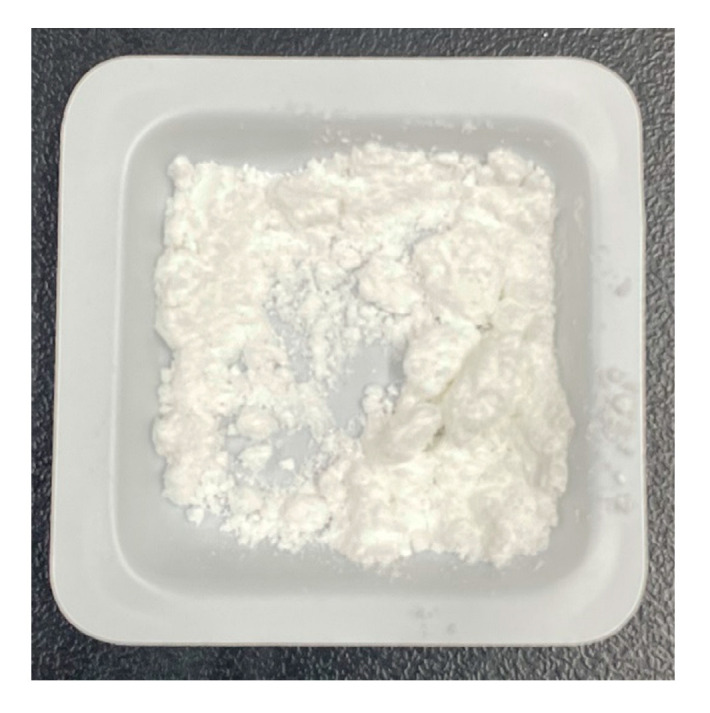
Urea.

**Figure 7:**
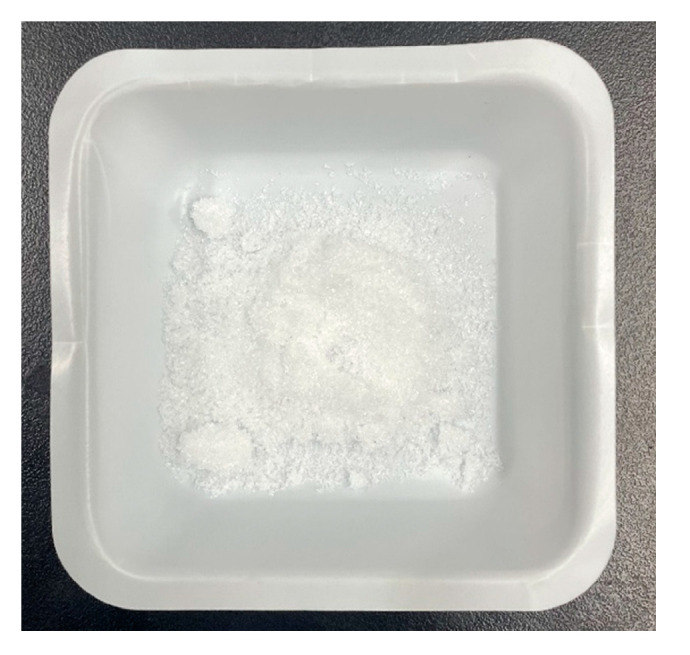
CTAC.

**Figure 8:**
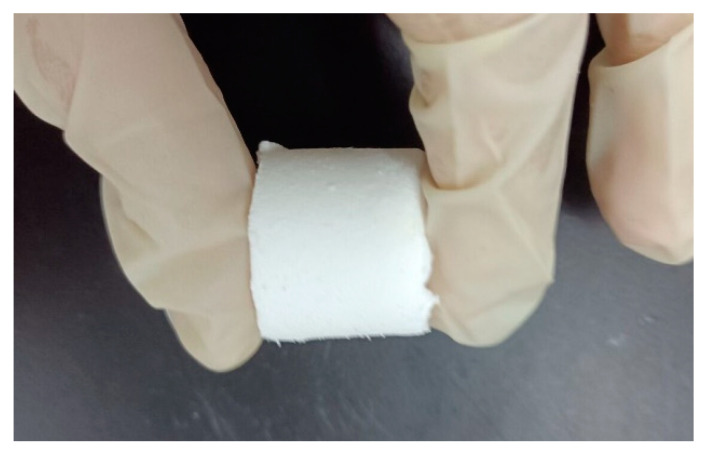
Silica aerogel.

The instruments used in the preparation process are a digital display temperature control magnetic stirrer and an electrothermal constant temperature blast drying box. The raw materials used include trimethoxymethylsilane, dimethyldimethoxysilane, deionized water, acetic acid, urea (Figure 6), and surfactant N-hexadecyltrimethylammonium chloride (CTAC) (Figure 7) [33]. The specific preparation process is as follows:

A total of 15 mL of trimethoxymethylsilane and dimethyldimethoxysilane in a volume ratio of 1:1 was mixed with 24 μL of acetic acid and 90 mL of deionized water in a beaker. Among them, acetic acid played a role in creating an acidic environment and promoting the hydrolysis reaction of silane. At the same time, 30 g of urea was added to promote the occurrence of the alkaline polymerization, and 4.8 g of surfactant (CTAC) was added for the catalytic hydrolysis reaction [34]. Under the 30 °C condition, the reaction was fully carried out by stirring for 30 min with a digital-temperature-controlled magnetic stirrer. After the stirring was completed, the silane mixture was poured into the mold, and the mold was sealed with a preservative film with a small hole. The mold used in this preparation was a PVC tube with a diameter of 20 mm, and each PVC tube was poured with about 30 mL of the above-mixed solution. It was then put into an electrothermal constant-temperature blast-drying box to form a silica gel, dried at 80 °C for 24 h, and initially formed a silica gel. At this time, the gel pores contained a certain amount of water, which needed to be replaced by a mixed solution of isopropanol and water at room temperature for 8 h. The mixed solution during the replacement process should ensure that the immersed gel block was fully solvent-exchanged. Since the replacement process required direct contact between the gel and the replacement solution, it was necessary to remove the aerogel from the container before the replacement, and the action should be as gentle as possible. After the isopropyl alcohol replacement was completed, the isopropyl alcohol solution was replaced with n-hexane to replace the isopropyl alcohol in the pore of the gel, and the replacement was continued at room temperature for 8 h, replaced three times; then, the gel was obtained after the replacement is completed. The silica wet gel had a certain morphology and strength, but it still needed to be dried to remove the remaining solvent in the pores, so that the pores were filled with air to complete the transformation of silica wet gel to aerogel. The bulk wet gel was placed in a drying oven and dried at 40 °C for 24 h. Finally, the silica aerogel was obtained as shown in Figure 8.

### 4.3. Characterization Analysis of Flexible Aerogel

The unique network structure and high porosity of silica aerogel also lead to their disadvantages such as low strength, poor toughness, poor hygroscopicity, and the easy destruction of nanoporous structures under external forces, which greatly limit their practical application. Therefore, overcoming the shortcomings of silica aerogel is of great significance for practical applications. To improve the mechanical properties of silica aerogel and expand its application range, many scholars at home and abroad have studied silica aerogel for many years [35]. Wu et al. used tetraethyl orthosilicate (TEOS) and methyltrimethoxysilane (MTMS) as silicon sources to prepare hydrophobic aerogels via supercritical drying and modification. The hydrophobic angle of the hydrophobic aerogel reached 140°, but the flexibility of the material was not mentioned. If silica aerogels are prepared via ambient-pressure drying, it is difficult to maintain the bulk structure of the aerogels, and their performance is also different from that of aerogels prepared via supercritical drying. Therefore, the preparation of flexible silica aerogels at a low cost and simple process is still a challenge.

Cheaper and less toxic silica precursors are preferred to reduce the cost of flexible silica aerogels and optimize the preparation process. Flexible hydrophobic aerogels prepared from methyltrimethoxysilane and dimethyldimethoxysilane can be completely restored to their original size after bending them to a certain extent and can be recycled, which is an ideal material for oil–water separation. After multiple adsorption–desorption cycles, the adsorption performance remained basically unchanged [36]. As a core part of filtration, aerogels can easily separate insoluble organic solvents from water. The prepared hydrophobic silica aerogel was dried in a drying oven at 80 °C to exclude the water in the sample preparation process to meet the requirements of subsequent characterization tests. The sample was labeled as SA for subsequent characterization tests.

#### 4.3.1. Surface Analysis

**Figure 9:**
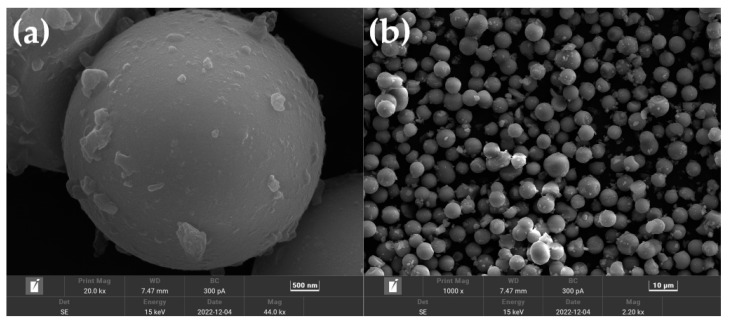
SEM image of silica aerogel.

A scanning electron microscope (SEM) is a high-resolution micro-analysis tool that can provide detailed information, such as the morphology, composition, and crystal structure of a material [37,38]. Figure 9 is the result of the morphology analysis of the sample using a scanning electron microscope. The two subgraphs (a) and (b) represent the SEM images of the same sample at different magnifications. It can be seen from the SEM images that the aerogel is mainly composed of methylsiloxane particles with a size of 2–3 μm and a large number of large pores (large pores of 890–1128 nm). It is speculated that the BET specific surface area is low, and the combination of low density and a large number of pores may make aerogel a good material for oil–water separation [39]. In addition, when an external force is applied to the aerogel, the pores between the particle chains create a large buffer space for Si–O–Si. The aerogel network is not easy to break, so the sample has good flexibility [40]. And because the methyl polarity on the Si–O–Si main chain is weak, the intermolecular force is reduced, and the flexibility is enhanced.

**Figure 10:**
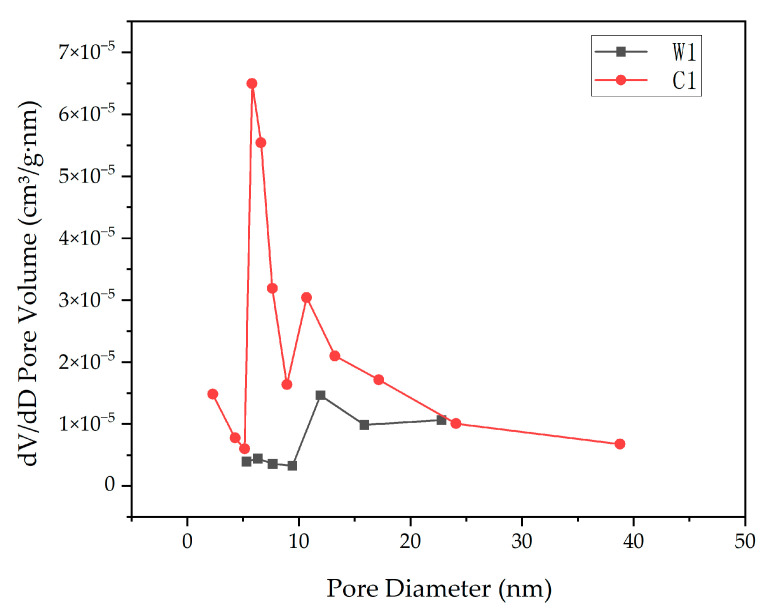
Pore size distribution.

In order to explore the internal pore size distribution of aerogels, we performed BET analysis on the aerogel samples, and the test results are shown in Figure 10. Sample C1 represents the aerogel after the diene reaction; Sample W1 represents the aerogel without the diene reaction. It can be seen that the pore size of the two samples concentrated in the range of 15 nm. At the same time, we also measured the BET surface area of Sample C1 as 1.0213 m^2^/g and the BET surface area of Sample W1 as 0.6021 m^2^/g. Combined with the SEM images of aerogels, it can be seen that there were no mesopores in the aerogel samples prepared in this project, and only macropores can be seen in the SEM images, which shows that the aerogel prepared in this study was a macroporous porous material.

#### 4.3.2. Water Contact Angle Analysis

**Figure 11:**
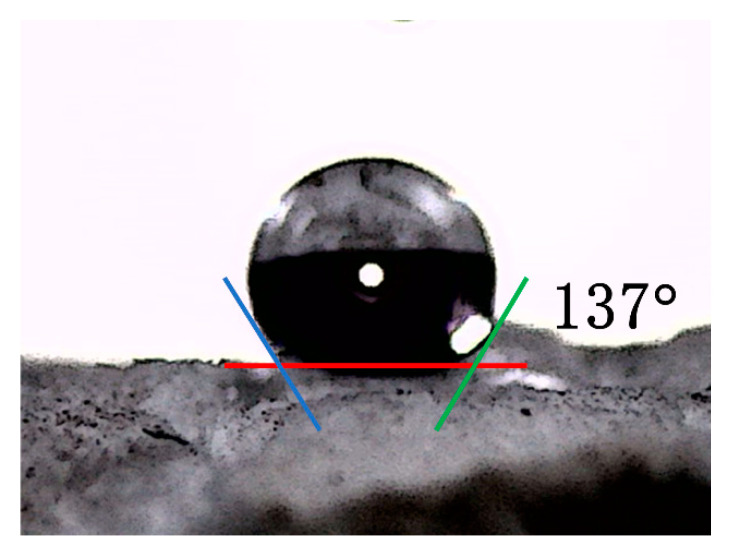
The water contact angle of silica aerogel.

Due to the large amount of hydrophobic methyl (–CH_3_) produced by MTMS and DMDMS, it is attached to the Si atom to form hydrophobic aerogels. It can be seen that water droplets formed on the surface of the sample, as shown in Figure 11, and the contact angle reached 137°. It showed superhydrophobic properties and provided a basis for subsequent oil–water separation. The superhydrophobic appearance is attributed to the rough surface’s synergistic effect generated by the sample surface’s porous structure and the surface hydrophobic methyl group, which was verified by subsequent FTIR spectroscopy.

## Figures and Tables

**Table 1:** The atomic percentage of samples treated under different conditions.

	Si (%)	O (%)	C (%)	O/Si	C/Si
SA-1	27.4	32.6	40	1.19	1.45

**Table 2:** Adsorption and separation rate of different oils by flexible aerogels.

Category *	Low-Rate Group Adsorption Separation Rate/mL·s^−1^	Medium-Rate Group Adsorption Separation Rate/mL·s^−1^	High-Rate Group Adsorption Separation Rate/mL·s^−1^
n-hexane	1.56	1.6	2.6
isopropanol	1.06	1.08	1.17
glycol	0.55	0.71	0.85

* Note: Due to the small volume of aerogel selected in the experiment, the adsorption and separation of high-viscosity vegetable oil took a long time, so they are not listed in the table.

## Data Availability

Not applicable.
